# Conservative Management of Trigeminal Neuralgia and Degenerative Cervical Myelopathy: A Case Report

**DOI:** 10.7759/cureus.55274

**Published:** 2024-02-29

**Authors:** Eric Chun-Pu Chu, Jamir P Rissardo

**Affiliations:** 1 Chiropractic and Physiotherapy Centre, New York Medical Group, Hong Kong, CHN; 2 Neurology Department, Cooper University Hospital, New Jersey, USA

**Keywords:** ‏facial pain, bell's palsy, oro-facial pain, trigeminal neuralgia, spinal manipulative therapy, chiropractor, chiropractic

## Abstract

It is hypothesized that degenerative cervical myelopathy (DCM) may induce or exacerbate trigeminal neuralgia (TN) through mechanisms such as direct compression of the spinal trigeminal tract, inflammation, or vascular issues, leading to ischemia within cervical segments C3-C4, where the spinal trigeminal nucleus extends. Here, we report the potential therapeutic impact of chiropractic treatment in a 55-year-old female with TN resistance to medical therapy and DCM. The patient received targeted chiropractic care, consisting of high-velocity, low-amplitude (HVLA) spinal manipulation of the C3-C7 and T1-T4 vertebral segments to address joint dysfunction, coupled with intermittent mechanical cervical traction for 20-minute sessions, and focused radial shockwave therapy aimed at myofascial trigger points within the trapezius and levator scapulae muscles. After initiating the chiropractic care plan, the patient experienced a significant reduction in the frequency and severity of TN pain, which persisted throughout the treatment period. Notably, this alleviation in symptoms was maintained at the six-month follow-up, suggesting a sustained therapeutic effect rather than a transient improvement. The lasting nature of the pain reduction provides a compelling argument for the long-term benefits of chiropractic intervention in the management of TN, particularly in cases with concurrent DCM.

## Introduction

Trigeminal neuralgia (TN) is a debilitating neuropathic disorder characterized by intense episodic facial pain, which is ascribed to the dysfunction of the trigeminal nerve responsible for the sensory innervation of the face, nose, mouth, scalp, eyes, and ears [1]. This condition, which typically manifests in middle- to older-aged adults, is acknowledged by the International Classification of Headache Disorders (ICHD) as a sharp paroxysm of pain in one or more divisions of the trigeminal nerve [2], with a global lifetime prevalence ranging from 0.03% to 0.3% [3]. While neurovascular compression of the intracranial portion of the trigeminal nerve has been frequently posited as a primary etiological factor, a significant number of cases remain idiopathic, with neurovascular compression absent in 4-89% of patients [4], hence casting attention towards alternative risk factors and the pathogenesis of TN.

Degenerative cervical myelopathy (DCM) is a common condition characterized by spinal cord compression, specifically within the cervical spine, which can lead to a spectrum of neurological symptoms. Recent literature underscores instances where patients with DCM undergoing spinal surgery experience remission of TN symptoms postoperatively, suggesting a potential role of cervical spinal pathology in the development of TN [5]. This is further corroborated by a substantial data review from a United States network that revealed that adults with TN were over 12 times more likely to have DCM than those without TN, indicating that DCM is a possible risk factor for TN [5].

The proposed link between TN and cervical spine pathologies is encapsulated in the hypothesis that DCM may induce or exacerbate TN through mechanisms such as direct compression of the spinal trigeminal tract, inflammation, or vascular issues, leading to ischemia within cervical segments C3-C4, where the spinal trigeminal nucleus extends [6-8]. These insights compel a reevaluation of the traditional understanding of TN, suggesting that in certain cases, the origin of TN could be traced to cervical cord compression or inflammation affecting the spinal trigeminal tract rather than neurovascular damage alone.

This study aimed to explore the potential link between TN and cervical spine pathologies and elucidate the role of DCM-related mechanisms in the pathogenesis of TN. By illustrating this connection, we hope to broaden the clinical perspective of TN, promote a more comprehensive diagnostic approach, and foster the development of targeted treatment strategies that address both the trigeminal nerve and cervical spine pathologies.

## Case presentation

A 39-year-old female housewife, sought chiropractic care for persistent left-sided facial pain, weakness in the left upper limb, and constant non-exertional chest pain at the sternum for nine months. The patient described the chest pain as a mild, constant, pressing discomfort located at the sternum. It was not provoked by physical exertion and did not exhibit diurnal variation in intensity. The discomfort was present most days, with the patient noting some episodic increases in severity that did not appear to be associated with any specific activities or triggers.

**Figure 1:**
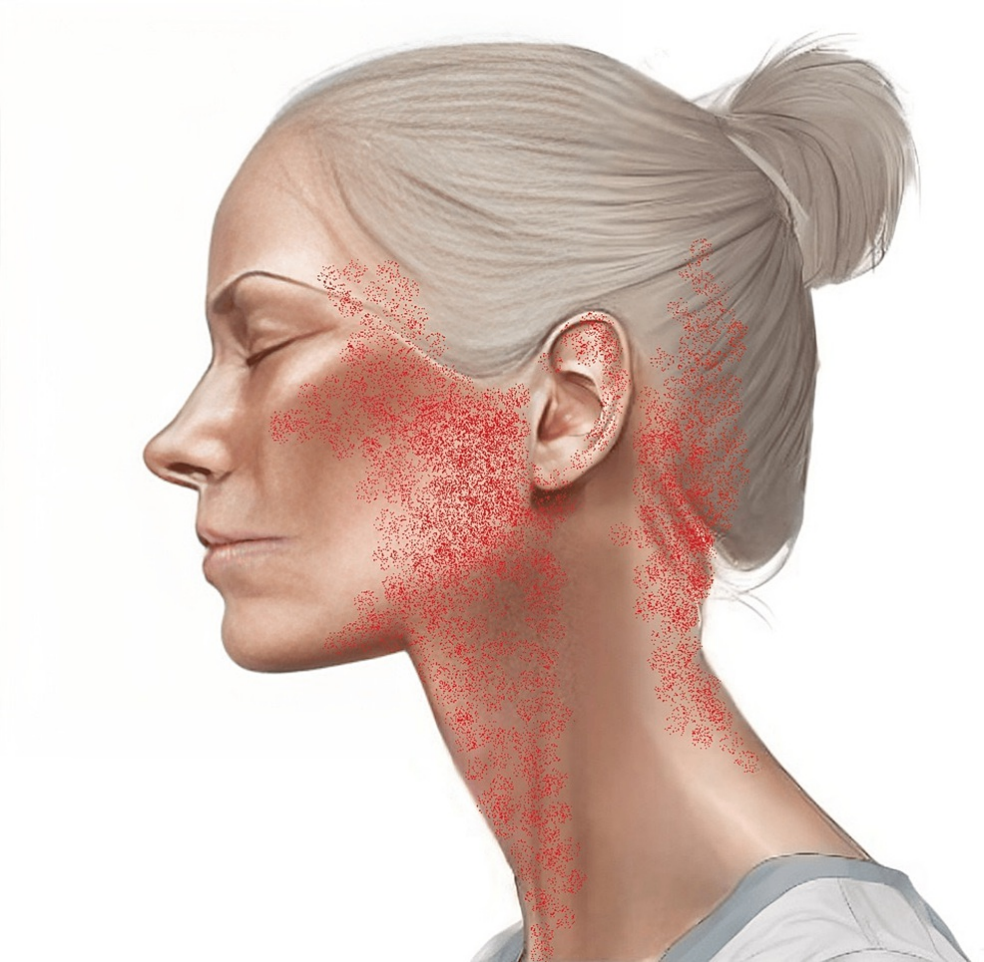
Illustration of the areas of facial pain The pain was described as a sharp sensation during swallowing and lateral neck flexion, originating in the left lateral head and ear and occasionally radiating toward the right ear Image Credit: Created by Eric Chu with the help of artificial intelligence (AI)

 Facial pain, described as a sharp sensation during swallowing and lateral neck flexion, originated in the left lateral head and ear and occasionally radiated towards the right ear (Figure 1). The patient reported bilateral neck pain that had been persistent over the same nine-month period as the left-sided facial pain. This condition was not a recent development but rather a chronic symptom that had been present alongside the facial discomfort. The neck pain was particularly noted upon awakening and had been a contributing factor to the patient's overall decrease in quality of life and contributing to depressive symptoms; however, the patient did not experience shoulder pain. The depressive symptoms were characterized by a persistent low mood, anhedonia, and a pervasive sense of hopelessness. Notably, sleep quality was unaffected.

The patient’s medical history revealed an episode of similar right-sided facial pain two years prior, which was exacerbated by tactile stimuli, such as tooth brushing, and was associated with gingival bleeding. The pain was localized in areas innervated by the trigeminal nerve. Subsequent dental evaluation led to the diagnosis and extraction of the right upper wisdom tooth, which was attributed to a dental cause of pain. She was then prescribed ibuprofen and antibiotics after the treatment.

Approximately nine months before presenting to our clinic, the patient began experiencing intermittent numbness in the left upper limb without paresthesia or neck pain and maintained a full range of motion. Concurrent dizziness without vertigo or diplopia had been reported previously. The symptoms evolved into daily episodes of left facial and scalp pain, along with a posterior headache, which intensified when speaking aloud. Dental assessments for gum swelling and pain, which are believed to be related to an impacted left lower wisdom tooth, were performed using anti-inflammatory drugs and antibiotics. Although the gum swelling resolved, facial pain persisted.

Subsequently, the patient experienced a gradual onset of fatigue and perceived weakness in the left upper arm, coupled with headaches exacerbated by prolonged periods of sedentary work. She reported upper neck pain reminiscent of torticollis, which was partially alleviated by massage therapy. Despite these interventions, the patient rated her pain as 3/10 and described it as a “string pulling” from the posterior neck to the head, suggesting muscular tension or nerve irritation.

Dental evaluations six months prior to the current presentation, prompted by jaw pain and cephalalgia, resulted in another tooth extraction, which failed to alleviate her symptoms. Extensive assessment in the emergency department three months prior, including a chest computed tomography scan, bloodwork, chest radiography, and electrocardiogram, had unremarkable findings and did not elucidate the cause of her persistent arm weakness and shoulder pain.

**Figure 2:**
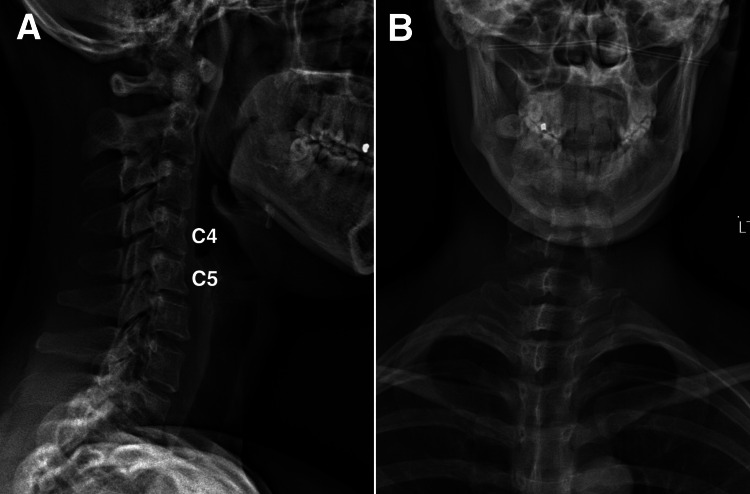
Radiograph of the cervical spine A) Reduced cervical lordosis and narrowing of the C4/5 disc were identified. B) Dextro-convexity at the lower cervical and thoracic regions was observed.

The patient’s medical history was notable for coronavirus disease 2019 (COVID-19) infection 12 months prior, which was followed by gastrointestinal discomfort managed with traditional Chinese medicine. Her cervical radiograph showed degenerative change (Figure 2). She also experienced ocular irritation, redness, and dryness due to long-term contact lens use, for which ophthalmological evaluation revealed no abnormalities. A family history of multiple sclerosis in the sister had also been documented.

A chiropractic examination revealed no evidence of limb weakness or facial asymmetry. Sensory, motor, and reflex examinations were normal. The patient complained of chest discomfort that had persisted for nine months. This discomfort was non-exertional and not associated with palpitations or other visceral disturbances. The patient’s general condition was good; she was ambulatory without assistance. Her pupils were equal, round, and reactive to light, with a score of 3+ bilaterally. The extraocular movements were normal, with no signs of nystagmus or diplopia. Cranial nerve function was grossly intact, and no cerebellar signs were observed. The cervical spine had a full range of motion except for a 10-degree restriction in extension. Palpation revealed tightness of the left upper trapezius and levator scapulae. Given the chronicity, severity, and characteristic nature of facial pain, trigeminal neuralgia was considered a differential diagnosis. Consequently, cervical spine magnetic resonance imaging (MRI) was performed to investigate persistent facial pain.

**Figure 3:**
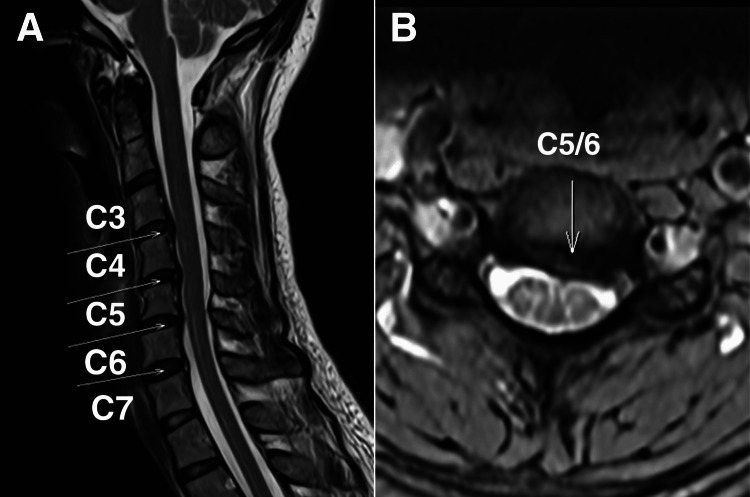
Cervical magnetic resonance imaging, T2-weighted sagittal view A) There is evidence of degenerative cervical myelopathy, including diffuse disc desiccation seen from C2/3 to C6/7 levels with mild loss of disc height at C5/6 level. C4/5 and C5/6 disco-osteophytic complex (white arrows) cause mild spinal canal stenosis and mild spinal cord deformation. B) C5/6 disco-osteophytic complex causes mild spinal canal stenosis and mild spinal cord deformation, more on the left (white arrow).

The cervical spine MRI revealed C5/6 spinal canal stenosis with mild deformation of the spinal cord, which was more pronounced on the left side (Figure 3). The patient was diagnosed with degenerative cervical myelopathy associated with trigeminal neurological symptoms.

The patient underwent a multimodal chiropractic therapy regimen, including spinal manipulation of the cervical and thoracic regions, intermittent mechanical cervical traction for 20-minute sessions, and shockwave therapy targeting the trapezius and levator scapulae muscles. Traction aims to relieve mechanical compression and promoting decompression of neural structures in the cervical spine could mitigate the contributing factors of DCM that may exacerbate TN symptoms. This form of traction was chosen with the intent to reduce intradiscal pressure and stretch soft tissues, which in turn may alleviate any impingement or irritation of the spinal trigeminal tract, potentially reducing the neuralgia experienced by the patient. Spinal manipulation focuses on C3-C7 and T1-T4 vertebral segments to address joint dysfunction.

The treatment was administered thrice weekly for two months. The patient experienced a significant reduction in symptoms within the first two weeks, and complete resolution of all symptoms was reported by the end of the two-month treatment period.

## Discussion

The confluence of TN and DCM is a nuanced paradigm for neuropathic pain management. Evolving evidence suggesting a bidirectional association between TN and cervical spinal pathology necessitates a reexamination of chiropractic interventions within this interplay [5]. Notably, chiropractic care, traditionally centered on biomechanical and functional restoration of spinal integrity [9,10], may have therapeutic potential beyond musculoskeletal ailments, extending into neuropathic conditions such as TN [11].

The differential diagnosis of TN, particularly in the presence of concurrent cervical myelopathy, presents a formidable clinical challenge. The non-specificity of orofacial pain symptomatology can confound the diagnostic process, leading to potential misdiagnosis or oversight of cervical etiologies [11]. A comprehensive evaluation, including detailed patient history, neurological and orthopedic examinations, and advanced imaging modalities such as MRI, is imperative to unravel the complexities of these coexisting conditions. Therefore, chiropractors’ diagnostic acumen must be commensurate with these complexities to discern the etiological nexus between TN and DCM.

Although the potential neuromodulatory effects of chiropractic therapy in ameliorating the aberrant nociceptive processing characteristics of trigeminal neuralgia have not been directly addressed in the current literature, cervical spinal cord stimulation has been deemed a secure and efficacious method for individuals with TN [12]. Spinal manipulative therapy (SMT) can also exert neuromodulatory effects that may alleviate atypical pain signal processing associated with TN. Additionally, adjuvant chiropractic interventions such as cervical traction, neuromobilization, and shockwave therapy could attenuate the spinal cord and nerve root compression implicated in DCM, thereby addressing a putative root cause of TN.

The clinical outcomes observed in this case report, derived from applying chiropractic modalities, support their consideration for treating complex neuropathic pain syndromes. However, chiropractors need to carefully appraise the risk-benefit profile of SMT, particularly in patients with advanced cervical spondylopathy, to obviate iatrogenic complications.

This case report has numerous implications for future clinical practice. First, it emphasizes the need for chiropractic practitioners to remain vigilant about neurological pathologies presenting as routine musculoskeletal complaints. Second, it reinforces the need for an integrated practice model where chiropractors collaborate with neurologists, pain specialists, and spine surgeons to manage patients with TN and DCM holistically.

Such interdisciplinary collaboration is paramount for formulating comprehensive treatment algorithms, which may include chiropractic care as adjunctive therapy. Ultimately, this integrative approach may enhance patient outcomes, foster innovation in treatment strategies, and spur further research into chiropractic management of neuropathic pain syndromes.

## Conclusions

The complex interplay between TN and cervical myelopathy challenges clinicians to broaden their diagnostic and therapeutic approaches by incorporating a comprehensive evaluation that considers potential cervical spine involvement. Integrating chiropractic care with non-invasive modalities has been demonstrated to potentially benefit patients with TN, particularly when cervical pathology is present. This case report advocates an interdisciplinary approach to patient management, emphasizing the importance of collaboration between chiropractors and other healthcare specialists. Further research on the chiropractic management of TN in the context of cervical myelopathy is needed to optimize patient outcomes and advance clinical practice.
